# IPSC-Derived Sensory Neurons Directing Fate Commitment of Human BMSC-Derived Schwann Cells: Applications in Traumatic Neural Injuries

**DOI:** 10.3390/cells12111479

**Published:** 2023-05-25

**Authors:** Kin-Wai Tam, Cheuk-Yin Wong, Kenneth Lap-Kei Wu, Guy Lam, Xiaotong Liang, Wai-Ting Wong, Maximilian Tak-Sui Li, Wing-Yui Liu, Sa Cai, Graham Ka-Hon Shea, Daisy Kwok-Yan Shum, Ying-Shing Chan

**Affiliations:** 1 School of Biomedical Sciences, Li Ka Shing Faculty of Medicine, The University of Hong Kong, Hong Kong, China; anthonykwtam@connect.hku.hk (K.-W.T.); yvonnecwcy0619@gmail.com (C.-Y.W.); kennethlkwu@gmail.com (K.L.-K.W.); u3506833@connect.hku.hk (G.L.); ltony@hku.hk (X.L.); wongwwt@hku.hk (W.-T.W.); tslimax@hku.hk (M.T.-S.L.); wingyuiliu@gmail.com (W.-Y.L.); caisa@hku.hk (S.C.); 2 Department of Orthopaedics and Traumatology, Li Ka Shing Faculty of Medicine, The University of Hong Kong, Hong Kong, China; gkshea@hku.hk; 3 State Key Laboratory of Brain and Cognitive Sciences, The University of Hong Kong, Hong Kong, China

**Keywords:** Schwann cells, human bone marrow stromal cells, sensory neuron, remyelination, directed differentiation, sciatic nerve injury, spinal cord injury

## Abstract

The in vitro derivation of Schwann cells from human bone marrow stromal cells (hBMSCs) opens avenues for autologous transplantation to achieve remyelination therapy for post-traumatic neural regeneration. Towards this end, we exploited human induced pluripotent stem-cell-derived sensory neurons to direct Schwann-cell-like cells derived from among the hBMSC-neurosphere cells into lineage-committed Schwann cells (hBMSC-dSCs). These cells were seeded into synthetic conduits for bridging critical gaps in a rat model of sciatic nerve injury. With improvement in gait by 12-week post-bridging, evoked signals were also detectable across the bridged nerve. Confocal microscopy revealed axially aligned axons in association with MBP-positive myelin layers across the bridge in contrast to null in non-seeded controls. Myelinating hBMSC-dSCs within the conduit were positive for both MBP and human nucleus marker HuN. We then implanted hBMSC-dSCs into the contused thoracic cord of rats. By 12-week post-implantation, significant improvement in hindlimb motor function was detectable if chondroitinase ABC was co-delivered to the injured site; such cord segments showed axons myelinated by hBMSC-dSCs. Results support translation into a protocol by which lineage-committed hBMSC-dSCs become available for motor function recovery after traumatic injury to both peripheral and central nervous systems.

## 1. Introduction

Peripheral nerve injury triggers the spontaneous reprogramming of Schwann cells to a reparative phenotype that scavenges myelin debris, supports axonal survival, and directs axonal regrowth across lesion gaps small enough for spontaneous recovery from injury [1]. Spontaneous repair becomes limited [2] when disruptive damage to the nerve exceeds the critical gap. Such cases can be remedied by bridging the stumps with nerve autografts or Schwann-cell-seeded axon guidance conduits that support the regeneration of axon–Schwann cell units across the severed tissue not only in the peripheral nervous system (PNS) [3,4,5], but also in the central nervous system (CNS) [6,7]. 

Given the lack of accessible Schwann cells from native tissues and the fate instability of Schwann cells derived in vitro, the clinical use of Schwann cell transplantation for tissue repair and remyelination has been limited. Primary Schwann cells that can be harvested from native tissues are limited by donor site morbidity, whereas Schwann-cell-like cells (SCLCs) derived from non-neural tissues [8,9] often display phenotypic instability and spontaneous dedifferentiation into myofibroblasts [10,11,12] unless steps are taken to promote commitment.

To this end, we derived a protocol whereby Schwann cells were derived from rats (pilot studies) and, subsequently, human bone marrow stromal cells (hBMSCs) [11,13,14], without the use of transforming viral vectors. A culture of hBMSCs in non-adherent conditions facilitated the expansion of neural stem/progenitor cells that were positive for nestin and glial fibrillary acid protein (GFAP) [15]. These cells were directed to differentiate into SCLCs with supplementation of glia-inducing factors (GIFs) in the culture medium [11,13,14]. Via the co-culture of SCLCs and embryonic rat dorsal root ganglia (DRG) neurons, contact-mediated Notch and ErbB signaling was found to direct the commitment of SCLCs to the Schwann cell fate [12]. However, the use of cross-species cells to generate SCLCs invites the risk of immune hypersensitivity and deters the use of such SCLC derivatives for transplantation therapy.

**Figure 1:**
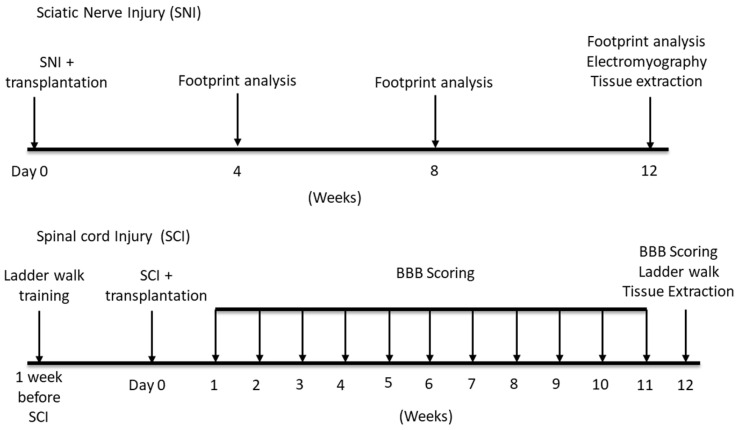
Timeline of the animal models.

To replace rat DRG neurons with a human counterpart for inducing the lineage commitment of SCLCs, we made use of a rapid single-step protocol to generate sensory neurons (hiSN) from human induced pluripotent stem cells (hiPSCs) [14]. In the current work, the co-culture of hSCLCs with hiSNs was found to mediate fate commitment of hBMSC-derived SCLCs to phenotypically stable Schwann cells (hBMSC-dSCs). The effectiveness of hBMSC-dSC transplants for remyelination therapy after neurotrauma was demonstrated in rat models of sciatic nerve injury (SNI) and spinal cord contusion injury (SCI) (Figure 1). 

In the SNI model, rats treated with nerve conduits that had been seeded with hBMSC-dSCs showed significant improvement in gait 12 weeks post-injury compared to null in the vehicle control. In the SCI model, co-treatment with hBMSC-dSCs and chondroitinase ABC (ChABC, which digest axon-restrictive chondroitin sulphate (CS) enriched in the glial scar of injured CNS tissue [7]) also resulted in significant improvement in motor function. The improvement in motor function was accompanied by robust axonal regrowth and remyelination, as observed in animals treated with hBMSC-dSCs. These results reinforce the use of hiSN in co-culture with hBMSC-derived SCLCs to provide for contact-mediated signaling towards fate-committed Schwann cells that promise translation into post-traumatic remyelination therapy. 

## 2. Materials and Methods

### 2.1. Isolation and Culture of hBMSCs

Human bone marrow samples were collected from the iliac spine of 5 healthy donors with use of syringe aspiration under sterile conditions. The study protocol was approved by the Institutional Review Board of The University of Hong Kong/Hospital Authority Hong Kong West Cluster (Study No. UW 10-157).

Stock samples of hBMSC were established with slight modification of the method of Wolfe et al. [16]. Briefly, bone marrow concentrate of each donor was diluted 5–10 times (as per sample quality) with BMSC medium, consisting of Minimum Essential Medium Eagle—Alpha Modification (MEM-alpha, Invitrogen, Waltham, MA, USA) with 15% (*v*/*v*) fetal bovine serum (FBS, French origin, Biosera, Nuaillé, France) and then plated onto a culture plate for maintenance in culture at 37 °C, 5% CO_2_, and 95% humidity for 10–14 days with medium changed on alternate days to remove suspending hematopoietic cells. Human BMSCs were harvested using TrypLE™ Express (Gibco, Roskilde, Denmark) and plated onto low-adherence 6-well culture plates (Greiner, Frickenhausen, Germany) at 50,000 cells/cm^2^ in BMSC medium. The cells were passaged at 1/3 density when they reached 80% confluence. Human BMSCs at passages 3 to 8 were sampled for immunocytochemical (ICC) assessment of markers CD73, CD90, CD105, and STRO-1.

### 2.2. Derivation of SCLCs from hBMSCs [5]

Briefly, hBMSCs in passages 3–6 were transferred to ultra-low-attachment 6-well culture plates (Corning, Corning, NY, USA) and cultured in sphere-forming medium (Neurobasal medium (NBM, Invitrogen, Carlsbad, CA, USA) supplemented with 2% B27 (Invitrogen, Carlsbad, CA,USA), 20 ng/mL epidermal growth factor (EGF, Peprotech, Rehovot, Israel), and 20 ng/mL basic fibroblast growth factor (bFGF, Peprotech, Rehovot, Israel). Neurosphere-like clusters emerged within 48 h and the cultures were maintained for 14 days. One third of the medium was replaced every 3 days with fresh sphere-forming medium that contained a triple concentration of supplements.

The hBMSC-derived neurospheres were transferred to 6-well culture plates (coated with laminin (Roche, Basel, Switzerland) and Poly-D-lysine (PDL, Sigma Aldrich, Saint Louis, MO, USA)) at 8–10 spheres/cm^2^, and cultured for 14 days in glia-induction medium (MEM-alpha medium supplemented with 10% FBS (South American origin, Biosera, France) and GIFs (5 μM forskolin (FSK, Sigma, St. Louis, MO, USA), 200 ng/mL β-heregulin (β-HRG, Peprotech, Rehovot, Israel), 5 ng/mL platelet-derived growth factor (PDGF)-AA (Peprotech, Rehovot, Israel), and 10 ng/mL bFGF)).

### 2.3. Derivation of Sensory Neurons (hiSNs) from hiPSCs [14]

Human iPSCs (IMR90) clone (#1) (WiCell Research Institute) was a gift from Prof. Elly S.W. Ngan (Department of Surgery, The University of Hong Kong). Briefly, hiPSCs were plated at a density of 1 × 10^5^ cells/well on Matrigel-coated 6-well plates and cultured for 8 days in SN differentiation medium (DMEM/F12 medium supplemented with 10% KnockOut Serum Replacement (KSR, Gibco), 1% penicillin/streptomycin (P/S, Thermo Fisher), 0.3 μM Retinoic acid (Sigma), and small-molecule inhibitors cocktail (SMIs: 0.3 μM LDN-193189 (Cellagen Technology, San Diego, CA, USA), 2 μM A83-01 (Cellagen Technology, San Diego, CA, USA), 6 μM CHIR99021 (BioVision, CA, USA), 2 μM RO4929097 (Cellagen Technology, San Diego, CA, USA), and 3 μM SU5402 (Tocris, Bristol, UK)). The medium was refreshed on alternate days. Neuron morphology was checked 6–8 days after induction. At this point, the medium was changed to SN maintenance medium (NBM supplemented with 10 ng/mL neurotrophin 3 (NT-3, R&D Systems, Wiesbaden, Germany), 20 ng/mL brain-derived neurotrophic factor (BDNF, R&D Systems, Minneapolis, MN, USA), 20 ng/mL nerve growth factor (NGF, R&D Systems, Minneapolis, MN, USA), 20 ng/mL glial cell line-derived growth factor (GDNF, R&D Systems, Minneapolis, MN, USA), and 1% P/S. The cells were further cultured for 14 days. 

### 2.4. Derivation of hBMSC-dSCs

The hBMSC-dSCLCs obtained were seeded (3000 cells/cm^2^) onto the hiSNs in culture and maintained in co-culture for 14 days in medium (MEM-alpha and NBM (1:1, *v*/*v*), supplemented with 5% FBS (South America origin), 1% (*v*/*v*) B27, 1% (*v*/*v*) P/S, 2.5 μM FSK, 2.5 ng/mL PDGF-AA, 5 ng/mL bFGF, 100 ng/mL β-HRG, and 5 ng/mL NGF). Half of the medium was replaced with fresh medium on alternate days. After 14 days, the cells were passaged to remove hiSNs. The culture was then maintained for at least 7 days in FBS-supplemented (10%) DMEM/F12 medium. 

### 2.5. In Vitro Myelination Assay of hBMSC-dSC [11]

**Figure 2:**
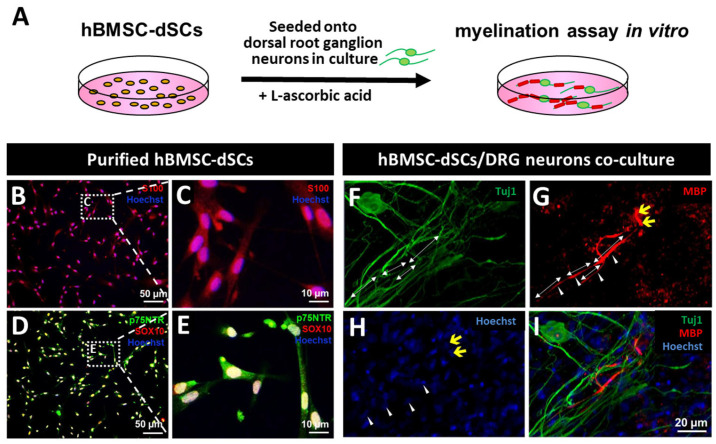
Myelination assay with hBMSC-dSCs in vitro. The co-culture model is illustrated in the schematic diagram (**A**). After co-culturing with hiSNs and removal of GIFs, the hBMSC-dSCs were passaged. The cells remained immuno-positive for Schwann cell markers, S100 ((**B**), enlarged in (**C**)), p75NTR, and SOX10 ((**D**), enlarged in (**E**)). When co-cultured with purified rat DRG neurons (Tuj1, (**F**)), hBMSC-dSCs myelinated the neurites resulting in MBP+ segments (**G**) along the DRG neuron network (panel (**I**)) (white double-headed arrows: myelin segments; white arrowheads: cell nucleus of hBMSC-dSCs (revealed in Hoechst stain (**H**)); yellow arrows: myelin segments on another neurite). Control neuron culture without hBMSC-dSCs confirmed absence of host Schwann cells (indicated by lack of p75NTR (**B**) and MBP (**C**) staining). Scale bars in (**B,D**): 50 μm; (**C,E**): 10 μm; (**F**–**I**): 20 μm.

To test the myelinating power of the derived cells on primary neurons other than iSNs, DRG neurons were extracted from Sprague–Dawley (SD) rat embryos of day 14.5–15.5. Cells were dissociated from the DRGs by digestion with TrypLE Express for 15 min at 37 °C. The dissociated DRGs were seeded at a density of 2–3 DRGs/cm2 on PDL/laminin-coated round coverslips (14-mm diameter, Thermo Scientific) and maintained in DRG medium (NBM supplemented with 2% B27 (*v*/*v*) and 20 ng/mL NGF). After 48 h, the DRG medium was further supplemented with 10 μM fluorodeoxyuridine (Sigma, Saint Louis, MO, USA) and 10 μM uridine (Sigma, Saint Louis, MO, USA) for removing highly proliferative endogenous glial cells and fibroblasts until pure neuron culture was obtained. The purified DRG neuron culture was maintained for another 7 days in DRG medium. We then seeded hBMSC-dSCs at a density of 8000 cells/cm^2^ onto the DRG neuronal network (Figure 2A) and maintained it with y co-culture medium (MEM-alpha and NBM (1:1, *v*/*v*), supplemented with 10% FBS (South American origin), 2% B27, and 10 ng/mL NGF). The medium was refreshed on alternate days. Until the alignment of hBMSC-dSCs with neurites was observed, L-ascorbic acid (50 μg/mL, Sigma, Heidelberg. Germany) was added to induce myelination. Myelination was assessed after 14 days of co-culture.

### 2.6. Rat Model of Sciatic Nerve Injury

All surgical procedures in this study were in strict accordance with the National Institute of Health Guidelines for Laboratory Animal Care and Safety, and with approved protocols by the Committee on the Use of Live Animals and Teaching and Research, the University of Hong Kong. Adult male SD rats (220–250 g) were anaesthetized with ketamine/xylazine mixture (Alfasan, Woerden, Netherlands, intraperitoneal injection). The skin on the left thigh was incised to expose the left sciatic nerve. At the femur level, a 10 mm segment of nerve was excised. At the two ends, 2 mm of the stumps was telescoped into a 16 mm long chitosan conduit so that a 12 mm gap was kept between the stumps. The conduit was filled with (i) hBMSC-dSCs (2 × 10^5^ cells in Matrigel) or (ii) Matrigel only (*n* = 4 each). Another group received reverse ligation with the excised fragment as a positive control (autograft). The conduit or autograft was secured with 7-O polydioxanone (PDS-II) monofilament suture (Ethicon). The wound was closed and the animals, after reviving in intensive care unit (Thermocare, CA), were returned to individual cages with food and water *ad libitum* for weeks with body weight monitored daily. Meloxicam (Boehringer Ingelheim, daily, in drinking water, one week after surgery), buprenorphine (Temgesic, Hull, UK, twice a day, subcutaneous injection, four days after surgery), oxytetracycline (Norbrook, Corby, UK, once every three days, subcutaneous injection, first week after surgery), and cyclosporin A (Selleckchem, immunosuppressant, 10 mg/kg, once per day, subcutaneous injection, throughout the holding period) were administered to the operated animals to suppress rejection. 

### 2.7. Sciatic Functional Index (SFI)

**Figure 3:**
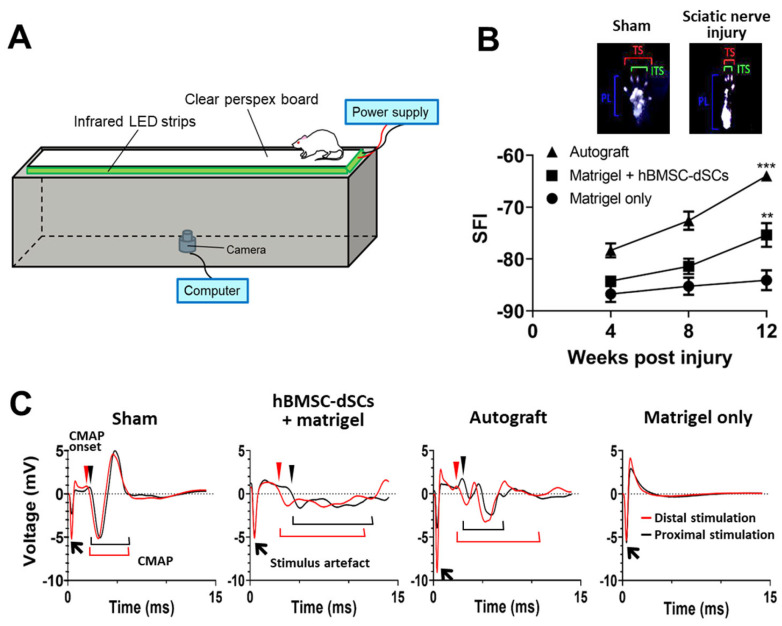
Restoration of sciatic function of injured leg. The sciatic function was assessed with sciatic function index (SFI). The footprints of injured and control legs were visualized with the infra-red recording setup as shown in panel (**A**). The contact of feet on the acrylic board generated white footprint due to frustrated total internal reflection (**B**). The operated side was longer in PL, and shorter in TS and ITS, when compared to the control side (PL: print length, indicated by blue brackets; TS: toe spread, indicated by red brackets; ITS: intermediate toe spread, indicated by green brackets). By week 12, SFI in rats that received autograft or grafts that contained hBMSC-dSCs were significantly improved compared to Matrigel-only controls (**B**). Representative sample traces of the recorded CMAPs are shown in (**C**). The signal patterns of proximal and distal end stimulations are indicated by black and red arrow heads, respectively. The onset of CMAP of proximal- and distal-end stimulation are indicated by black and red arrowheads, respectively. ** *p* < 0.01, *** *p* < 0.001. *n* = 4 for each animal group.

The sciatic nerve provides motor input to the lower limbs, including the feet. One of the most measurable movements is the spread of toes, as controlled by the peroneal nerve, a distal branch of the sciatic nerve. Sciatic functional index, one of the gold standards for assessing functional recovery of sciatic nerve [17,18], was used. Functional recovery was monitored based on footprint analysis conducted every four weeks after surgery. An infrared walkway system was used for collecting the footprint data. The construction of the system was based on Fricker et al. [19] with modifications (Figure 3A). The footprints revealed on the acrylic board surface due to frustrated total internal reflection were recorded with infra-red camera with a resolution of 1920 × 1080 at 30 frames/second. For each animal, 3 footprints that were made during steady walking were assessed using ImageJ software (NIH). 

Three parameters were measured (Figure 3B): (i) the print length (PL, distance from the third toe to the heel), (ii) the toe spread (TS, distance from the 1st to the 5th toe), and (iii) the intermediate toe spread (ITS, distance from the 2nd to the 4th toe). The data obtained were used to compute the following three factors:

$$\;\left(i\right)\;Print\;length\;factor\;\left(PLF\right)\;=\;\frac{Experimental\;PL\;-\;NormalPL}{Normal\;PL}$$

$$\left(\mathrm{ii}\right)\;Toe\;spread\;factor\;\left(TSF\right)\;=\;\frac{Experimental\;TS\;-\;NormalTS}{Normal\;TS}$$

$$\;\left(\mathrm{iii}\right)\;Intermiediary\;toe\;spread\;factor\;\left(ITSF\right)\;=\;\frac{Experimental\;ITS\;-\;Normal\;ITS}{Normal\;ITS}$$

The SFI was calculated by adapting the above factors into the following formula [17,18]:

SFI = −38.3 × PLF + 109.5 × TSF + 13.3 × ITF − 8.8

### 2.8. Electromyography

For measuring electromyography, the rats were anaesthetized with inhalation of isoflurane (1–2%) and kept warm at 37 °C. The sciatic nerve and gastrocnemius muscle on both sides were exposed. The intact nerves of the contralateral side were taken as the control. Double-hooked stainless steel stimulating electrode was used to suspend and stimulate the sciatic nerve at the proximal end (the end close to the spinal cord) or the distal end (the end close to gastrocnemius muscle), at 2 mm each from the graft. Two stainless-steel needle electrodes were inserted to gastrocnemius muscle for recording the compound muscle action potentials (CMAPs). The nerves were stimulated with single electrical pulses at supramaximal intensity. Similar procedures and measurements were made on the contralateral, intact side of each rat. 

### 2.9. Rat Model of Spinal Cord Contusion 

Adult male SD rats (220–250 g) were anesthetized by intraperitoneal injection of ketamine/xylazine cocktail (Alfasan). The spinal cord was exposed by dorsal laminectomy of the T9–T10 vertebrae. The contusion injury was induced by a weight-drop impactor device (W.M. Keck Center for Collaborative Neuroscience, Rutgers, State University of New Jersey). The animals were randomly assigned to two groups for contusive spinal cord injury, either moderate injury (dropping 10 g weight from 25 mm height) or severe injury (dropping 25 g weight from 50 mm height) [20,21]. To assess the success of contusion, we checked if (i) hemorrhage at the lesion site of the exposed spinal cord was observed immediately after contusion, and (ii) the BBB score at week one post-surgery was ≤2.

One of the following five treatments was administered to the operated rats: (i) Chondroitinase ABC (ChABC) injection followed by hBMSC-dSCs transplantation, (ii) ChABC injection followed by transplantation of human Schwann cells (hSCs, ScienCell, Catalogue #1700, passage 5 to 6), (iii) hBMSC-dSCs transplantation only, (iv) ChABC injection only, and (v) PBS injection only. ChABC was injected to the rostral and caudal edges of the lesion site. To each site, 50 mU ChABC (in 2 μL PBS) was injected. The grafted cells (hBMSC-dSCs or hSCs) were injected to 1 mm anterior and 1 mm posterior to the lesion site (for each injection, 2 × 10^5^ cells in 2 μL MEM-alpha). The muscle and skin were sutured with 4-0 PDS-II suture and 4-0 nylon sutures, respectively. Post-operative care was similar to that for the sciatic-nerve-injured rat. Manual expression of urinary bladders was performed at least twice a day until recovery of the micturition reflex.

### 2.10. Basso, Beattie, and Bresnahan (BBB) Score Test 

The BBB locomotor rating scale [22] was used for the evaluation of locomotor recovery of rats with spinal cord injury. Hindlimb movement was scored from 0, indicating no observable spontaneous movement, to 21 points, indicating normal locomotor movements. Assessments were made weekly for up to 12 weeks post-operation by placing the rats individually onto a circular open field with a 100 cm diameter. The rats were encouraged to explore the open field for 4 min and the process was videotaped. The average score from two independent experimenters blinded to the experimental groups was considered the final score.

### 2.11. Ladder Walk Test

Before operation, the rats were exposed to the testing environment for training. At 12 weeks post-operation, the animals were encouraged to cross the ladder from a neutral cage to their home cage. The ladder was elevated at an angle of 55°. The whole process throughout the test was videotaped. The evaluation of hindlimb placement was performed according to the 7-category scaled foot fault scoring by Metz and Whishaw [23]. 

### 2.12. Immunocytochemistry (ICC)/Immunohistochemistry (IHC)

For ICC, the cultures were fixed with 4% formaldehyde. hBMSC-derived neurosphere-like clusters were pelleted and resuspended in PBS and cytospun onto Superfrost slides (Thermo Scientific), followed by fixation with 4% formaldehyde. The cultures and cell clusters were then permeabilized and blocked in PBS with 0.1% Triton-X 100 and 3% bovine serum albumin (BSA). 

For IHC, the rats were sacrificed at 12 weeks post-surgery and perfused with 0.9% saline followed by 4% formaldehyde. The sciatic nerves and spinal cords were extracted and post-fixed in formaldehyde for 2 h at room temperature. Fixed sciatic nerves and spinal cords were incubated in 30% sucrose at 4 °C for 4 days and then sectioned to 20 μm using CryoStar™ NX50 Cryostat (Thermo Scientific). The fixed sections were washed with PBS and then permeabilized and blocked in PBS with 0.7% Triton-X 100 and 3% BSA. 

The samples were probed for CD73 (mouse, BD Pharmingen, 1:400), CD90 (mouse, BD Pharmingen, 1:500), CD105 (mouse, BD Pharmingen, 1:400), STRO-1 (mouse, R&D Systems, 1:400), nestin (mouse, R&D Systems, 1:400), CD45 (mouse, Millipore, 1:400), SOX2 (mouse, R&D Systems, 1:200), SOX10 (mouse, R&D Systems, 1:200), SSEA4 (mouse, DSHB, 1:400), Tuj1 (mouse, Biolegend, 1:400 (ICC) 1:200 (IHC); rabbit, Biolegend, 1:400 (ICC) 1:200 (IHC)), NF200 (NF200, rabbit, Sigma, 1:100), p75^NTR^ (rabbit, Abcam, 1:500 (ICC) 1:200 (IHC)), myelin basic protein (MBP) (rabbit, Abcam, 1:200 (ICC); mouse, Millipore, 1:100 (IHC)), S100β (rabbit, Dako, ready-to-use), and human nucleus (mouse, Millipore, 1:50). Immunopositive signals were revealed with anti-mouse IgG Alex488/Alexa594 and anti-rabbit IgG Alex 488/Alexa 594 (Goat, Life Technologies, 1:400). The cell nuclei were counterstained with Hoechst 33,258 (Sigma). Fluorescent images were captured with LSM 800 Confocal microscope (Carl Zeiss).

### 2.13. Transmission Electron Microscopy

Formaldehyde-fixed tissues were trimmed into 1 mm cubes and sent to the Electron Microscope Unit, the University of Hong Kong, for further tissue processing. In brief, the tissue blocks were treated with cacodylate buffer with 0.1 M sucrose, then post-fixed in 1% osmium tetroxide for 1 h at room temperature. The tissue blocks were dehydrated in ethanol stepwise from 50% to 100% and then embedded in epoxy resin. The samples were sectioned to 75–90 nm thick with glass/diamond knife using ultra-microtome and then mounted on 150 mesh hexagonal copper grids. After staining with 2% aqueous uranyl acetate for 20 min, images were captured under a Philips CM100 transmission electron microscope.

### 2.14. Statistical Analysis

One-way analysis of variance (ANOVA) was used for the evaluation of significant differences between endpoint scores followed by Tukey’s *post hoc* test. Two-way ANOVA followed by Tukey’s post hoc test was used to evaluate the significant differences based on dpi and treatment group. Poisson regression (log–linear model) was used to evaluate the difference in foot fault scores. Differences were considered statistically significant with * *p* < 0.05, ** *p* < 0.01, *** *p* < 0.001, and **** *p* < 0.0001.

## 3. Results

### 3.1. Characterization of hBMSC-dSC

Bone marrow samples were collected from healthy donors and the cell contents were expanded in vitro. By passage 3, the purity of the BMSCs reached ≥95.08 ± 5.83% without the contamination of hematopoietic stem cells, as evidenced by the positive immunostaining of BMSC but the lack of hematopoietic stem cell markers (Table S1, Figures S1 and S2A). Over half of the cells expressed nestin, the neural stem cell marker, affirming the presence of neural crest cell derivatives among the hBMSCs. Following non-adherent neurosphere-forming culture (Figures S2B and S3), the hBMSC-derived neurospheres were transferred to an adherent culture plate and treated with GIFs to induce SCLC differentiation (Figure S2C). After co-culture (Figure S2F) with hiSNs (Figures S2E and S4C) derived from hiPSCs (Figures S2D and S4A,B), 90% of the cells were positive for Schwann cell markers (S100, 92.14 ± 1.43%; SOX10, 89.55 ± 2.06%; p75NTR, 90.94 ± 5.66%) (Figure 2B–E and Figure S2G). The hBMSC-dSCs maintained high expression of these markers and did not turn into myofibroblasts even after the withdrawal of GIFs in subcultures where hiSN failed to survive. The marker expression and cell morphology could be kept for more than 12 population-doubling periods. In contrast, the SCLCs which had not been subjected to co-culture with neurons adopted fibroblast-like morphology following the withdrawal of the glial induction factors within two weeks, reminiscent of observations in our previous publication. [11] Human BMSC-dSCs myelinated primary DRG neurites (Figure 2F–I, also in Figure S5) in co-culture, thus demonstrating the functionality of hBMSC-dSCs in vitro. 

### 3.2. Motor Function Recovery after Transplantation

To test for *in vivo* function of hBMSC-dSCs in promoting functional recovery after PNS or CNS trauma, sciatic nerve injury and spinal cord contusion models were established in rats.

#### 3.2.1. Sciatic Nerve Injury Model

Regrowth of the transected sciatic nerve in the left leg of SD rats across a critical gap length through an hBMSC-dSC-loaded chitosan conduit, as previously described [3]; this was used to evaluate effectiveness of hBMSC-dSCs in promoting axonal regrowth. An autograft with reverse ligation of the transected segment was used as the positive control, while negative controls received conduits loaded with Matrigel only. While all groups showed progressive improvements post-operation (Figure 3B), the autograft group and the hBMSC-dSC-transplanted group performed significantly better than the Matrigel-only group in SFI by 12 weeks post-lesion. Improvement in Matrigel-only group (*n* = 4) from week 4 (SFI: −86.75 ± 3.07) to week 12 (SFI: −84.10 ± 3.84) was not statistically significant (*p* = 0.32). The SFI of the hBMSC-dSC-transplanted group (*n* = 4) improved significantly from -84.25 ± 2.05 in week 4 to −75.37 ± 4.55 in week 12 (*p* < 0.01), while the autograft group (*n* = 4) improved from −78.34 ± 2.69 to −63.98 ± 2.08 (*p* < 0.001). The electromyographic pattern (Figure 3C, *n* = 2) also demonstrated that electrical signals were transmitted through the hBMSC-dSC graft to the gastrocnemius muscle in the hBMSC-dSC-transplanted group in contrast to the nil response observed in the Matrigel-only group. 

#### 3.2.2. Spinal Cord Injury Model

We demonstrated that Schwann cells were capable of myelinating regrowing axons in the hemisected spinal cord of rats [7]. To test if hBMSC-dSCs likewise mediate recovery in an environment containing myelin debris, we adopted the spinal cord contusion model with two levels of severity (moderate and severe), performed according to Verma et al. [21]. A group transplanted with primary human Schwann cells served as a control. This type of cell source has been proven to be capable of remyelinating marked axons, though it is not clinically accessible. In addition to peri-lesional hBMSC-dSC transplants, ChABC was injected to the edge of the contusion site to remove the CS-rich axon-restrictive barrier formed in response to CNS neural trauma and when astrocytes confront Schwann cells [7,24,25]. 

**Figure 4:**
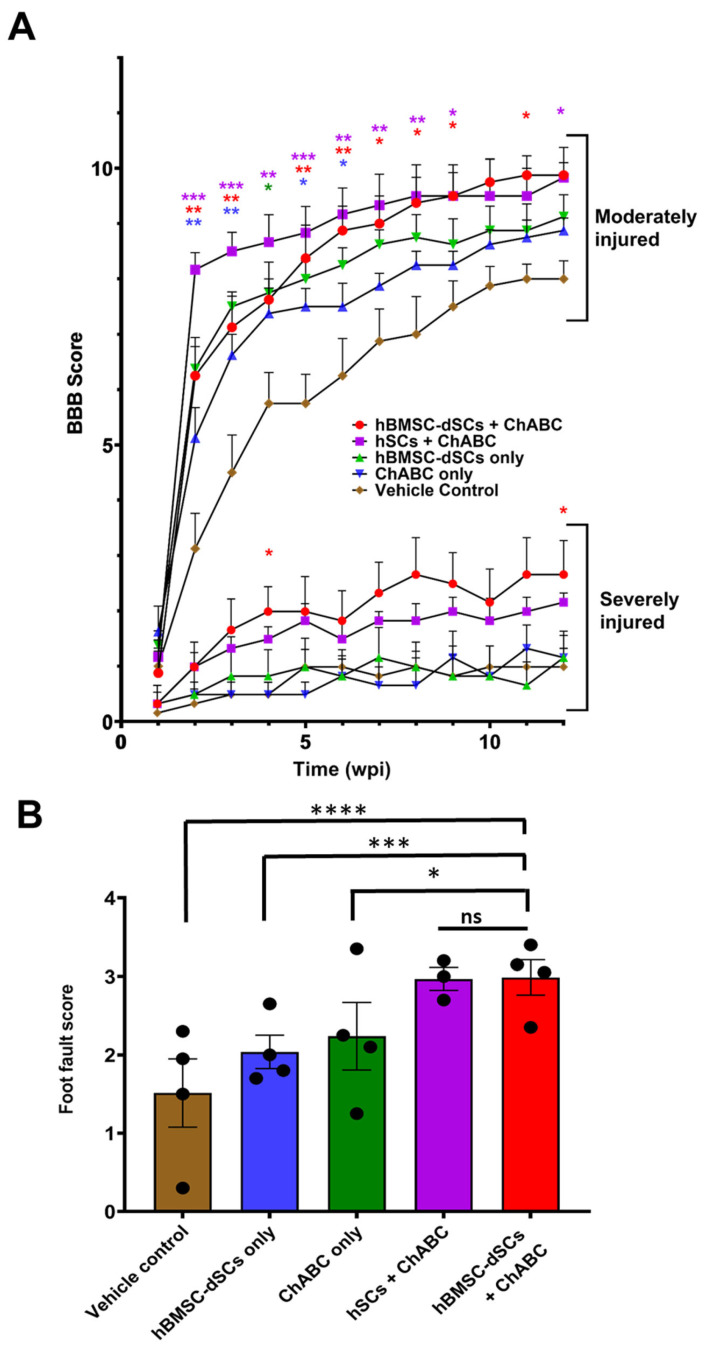
Behavioral tests after spinal cord injury. BBB scores of rats with moderate or severe spinal cord contusive injury are shown in panel (**A**). Statistically significant improvement in the treatment groups versus the vehicle control group are color-coded. Foot fault scores in the ladder walk test for rats with moderate spinal cord injury are shown in panel (**B**). wpi = week post-injury * *p* < 0.05, ** *p* < 0.01, *** *p* < 0.001, **** *p* < 0.0001, *n* = 4.

As expected, the improvement in motor function as reflected by the BBB score of rats receiving hBMSC-dSCs or primary hSCs alone was not significantly different from that of the control group (Figure 4A). However, when hBMSC-dSCs were transplanted with a concurrent application of ChABC to remove the restrictive CS-rich barrier around the injured site, rats showed significant improvement in BBB score, especially in moderate injury (9.88 ± 0.62) (Figure 4A). Similar results were observed for the ladder walk test (Figure 4B). Rats co-transplanted with Schwann cells (both hSCs and hBMSC-dSCs) and ChABC also performed significantly better in foot fault scores than other groups. 

### 3.3. Myelination in Axons That Grew across the hBMSC-dSC-Filled Lesion Gap in Sciatic Nerve

**Figure 5:**
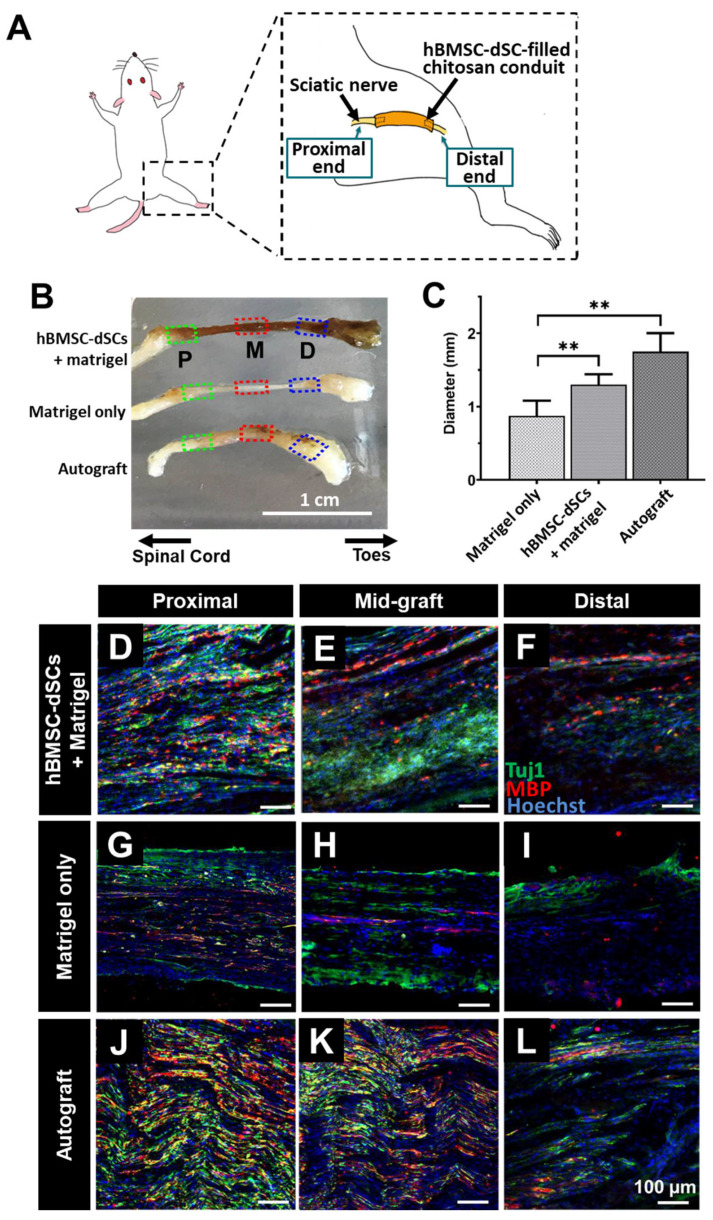
Chitosan conduit bridging a sciatic nerve gap. A conduit was grafted in replacement of the excised nerve segment (**A**). The 12 mm gap between the stumps was bridged with a conduit filled with Matrigel or hBMSC-dSCs suspended in Matrigel. As a positive control, the excised nerve segment was reversely ligated to the stumps. Regenerated nerve segments were harvested at 12 weeks post-lesion (**B**). From top: Matrigel with hBMSC-dSCs (**D**–**F**), Matrigel-only (**G**–**I**), and autograft (**J**–**L**). “Proximal”, “mid-graft”, and “distal” regions of the nerve segments are labeled as “P”, “M”, and “D”, respectively. (**C**) The diameter of the mid-graft region was measured (*n* = 4). The segments were sectioned and immunostained for axons (Tuj1) and myelin (MBP). Scale bars in (**D**–**L**): 100 μm. ** *p* < 0.01, versus “Matrigel-only” group.

To further confirm that the enhanced behavioral recovery was mediated by the regeneration of the transected sciatic nerves in rats treated with hBMSC-dSC, the regenerated sciatic nerves were harvested for histology 12 weeks post-injury. After carefully removing the chitosan conduit, we measured the mid-graft diameter of the regenerated sciatic nerve segment within the lesion gap (Figure 5A–C). The diameters of the regenerated segments in the autograft group and the hBMSC-dSC group were significantly thicker than those of the controls. Notably, no statistically significant difference in diameter between the hBMSC-dSC and autograft groups was found. Immunostaining for axon fibers (Tuj1+) and myelin (MBP) within the regenerated segment (Figure 5D–L) demonstrated that axons reached the distal end in both the autograft and hBMSC-dSC groups. Substantial MBP signals were observable at the distal end of both groups, though they were not as high as those at the proximal end. Thus, myelinated axons were restored in both groups, indicative of a more mature state of regeneration. On the other hand, while Tuj1-positive axon fibers were also detected in the distal end for the Matrigel-only group, an MBP signal was barely detected. This suggests that the progress of neuronal regeneration was accelerated in the presence of hBMSC-dSCs.

**Figure 6:**
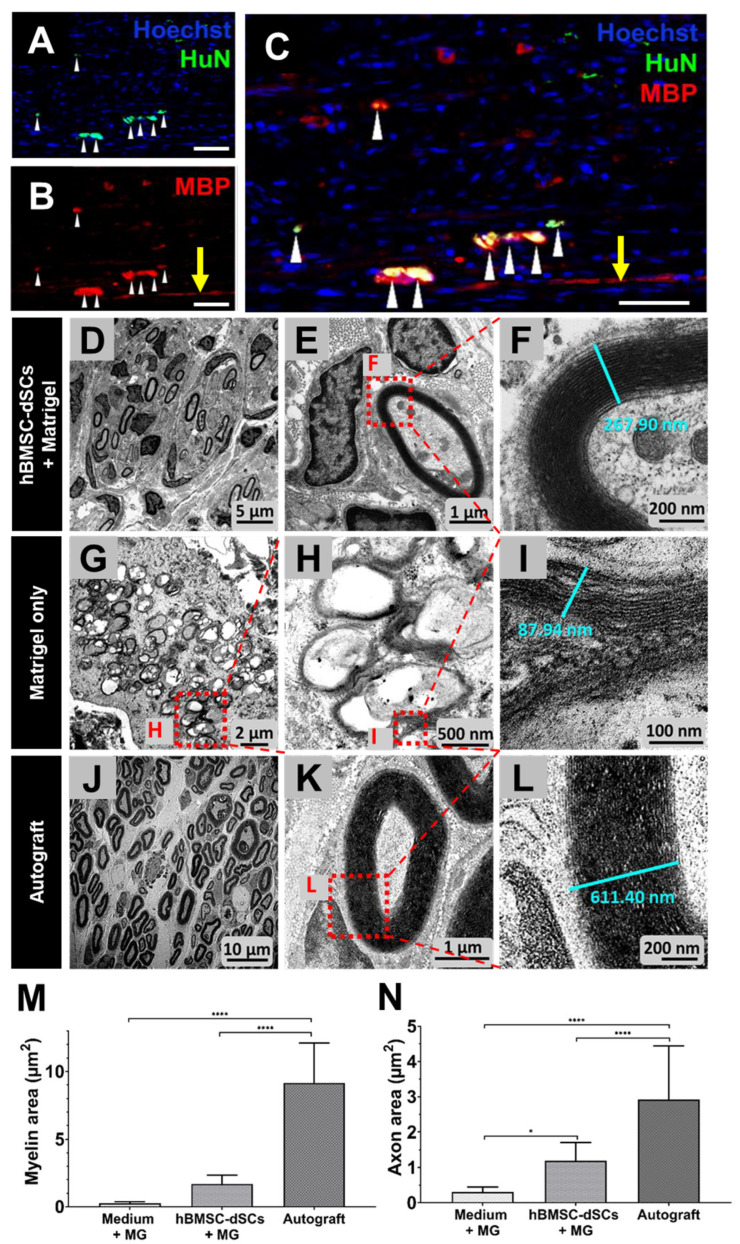
Regenerating nerve three months after sciatic nerve injury. Longitudinal sections of the regenerating nerve were stained for human nuclei (HuN, (**A**)) to track the hBMSC-dSCs. The images shown were captured from the mid-graft region. Sites doubly positive for HuN and MBP (**B**,**C**) indicated myelination by the hBMSC-dSCs (white arrowheads). The yellow arrows indicated myelination by endogenous Schwann cells. The nuclei of all cells in the specimen were Hoechst counterstained. Scale bars: 50 μm. TEM images were captured from mid-graft region of regenerating sciatic nerves. From top: Matrigel with hBMSC-dSCs (**D**–**F**), Matrigel-only (**G**–**I**), and autograft (**J**–**L**). Numbers in (**F**,**L**,**I**) marked the width of typical myelin in the regenerating nerve. For each sample, 4 random visual fields were taken, and the myelin area (**M**) and axon area (**N**) were measured. * *p* < 0.05, **** *p* < 0.0001.

To deduce if the myelination observed was due to transplanted hBMSC-dSCs or endogenous Schwann cells, we stained the sections for human nucleus antigen (HuN), which was only expressed by hBMSC-dSCs in the sample. Both HuN+ and HuN- cells expressed MBP within the graft, suggesting that both endogenous Schwann cells and hBMSC-dSCs contributed to the myelination observed (Figure 6A–C). However, almost all HuN+ cells were also MBP+, suggesting that the transplanted hBMSC-dSCs actively participated in the myelination of the regenerating axons. 

The quality and structural integrity of myelination in the mid-graft region was visualized by TEM. Morphometric analysis of the micrographs revealed thicker myelin in the hBMSC-dSCs transplanted graft (Figure 6D–L), but the difference in myelin area between the Matrigel-only group and hBMSC-dSCs group was not statistically significant (Figure 6M). The total cross-sectional area of axons, another indicator of nerve regeneration, was significantly larger in the hBMSC-dSC group compared to the Matrigel-only group (Figure 6N). The regenerated axons in rats that received hBMSC-dSC-loaded grafts were organized into fascicles as in the normal peripheral nerve (Figure 6D; the fascicles are outlined in Figure S6).

### 3.4. Regeneration of Myelinated Axons across Lesioned Spinal Cord Required Both hBMSC-dSCs and ChABC

**Figure 7:**
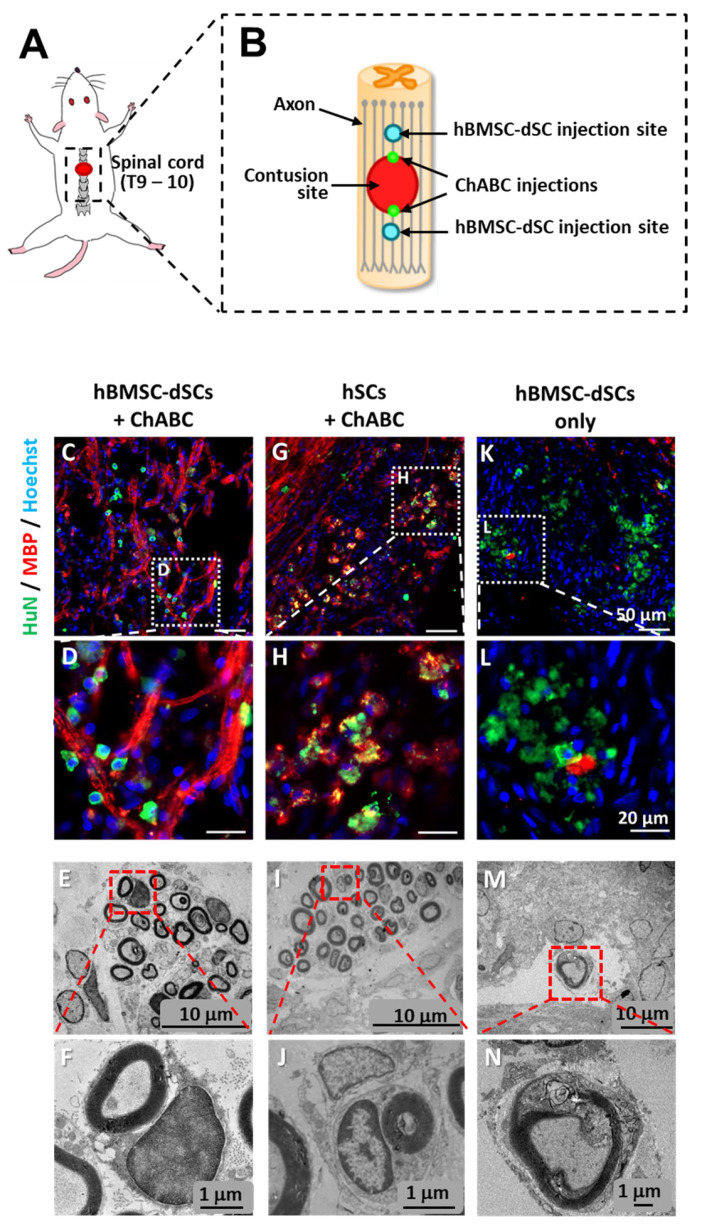
*Spinal cord injury and remyelination after hBMSC-dSC transplantation.* A schematic diagram showing respective sites of thoracic cord injury and micro-injections of hBMSC-dSCs and ChABC (**A**,**B**). Representative images of the lesion site captured from rats that received hBMSC-dSC transplantation and ChABC treatment (**C**,**D**); hSC transplantation and ChABC treatment (**G**,**H**); hBMSC-dSC transplantation only (**K**,**L**). Hoechst, HuN, and MBP staining to identify transplanted hBMSC-dSCs and myelination. Cord sections as examined under TEM: hBMSC-dSCs and ChABC treatment (**E**,**F**); hSCs and ChABC treatment (**I**,**J**); hBMSC-dSCs treatment only (**M**,**N**). Scale bars for (**C**,**G**,**K**): 50 μm; (**D**,**H**,**L**): 20 μm; (**E**,**I**,**M**): 10 μm; (**F**,**J**,**N**): 1 μm. *n* = 5.

In the spinal cord contusion model (Figure 7A,B), HuN immunopositive cells were detected within the spinal cord lesion of all Schwann-cell-transplanted groups (hBMSC-dSC-only, hSCs + ChABC, and hBMSC-dSCs + ChABC) (Figure 7C,D,G,H,K,L, also in Figure S8). However, MBP positivity was only observed in the groups that received cell transplant and concurrent application of ChABC. Staining for CS epitope (CS56) showed that the CS content at the lesion boundary was lower in the ChABC-treated rats compared to the controls (Figure S7). This result was confirmed with the immunopositivity of the CS stub (produced after CS digestion) at the ChABC injection site. This suggests that the removal of CS moieties by ChABC was a prerequisite for the SC-mediated remyelination of axons that grew across the lesion site. Analysis of the TEM images of transverse sections of the lesion site (Figure 7E,F,I,J,M,N) confirmed compact myelin in the groups receiving Schwann cells and/or ChABC treatment. The highest number of myelinated axons was observed in the groups treated with both ChABC and Schwann cells, congruent with the superior behavioral recovery of these groups.

## 4. Discussion

In this study, we made use of hiSNs to mediate the commitment of hBMSC-SCLCs to the Schwann cell fate. The resultant hBMSC-dSCs were fate stable for multiple passages even after the withdrawal of GIFs and neurons. These hBMSC-dSCs were capable of myelinating axons both in vitro and in vivo. We further demonstrated the efficacy of promoting functional recovery following the transplantation of hBMSC-dSCs into sites of traumatic neural tissue injury as exemplified by sciatic nerve injury and spinal cord injury. Histological results revealed that these cells enwrapped regrowing axons with compact myelin in both PNS and CNS. Transplantation of hBMSC-dSCs led to significant improvement in motor function in the sciatic nerve injury model, reaching scores that approached those of autografts, the gold standard for bridging critical nerve gaps. When the restrictive CS moiety in the glial scar around the spinal cord lesions [24,26] was removed via ChABC activity, hBMSC-dSCs further demonstrated the capability of mediating axonal regrowth in the contused spinal cord; as a result, improvement in motor performance similar to that due to the transplantation of primary human Schwann cells was observed. Results open avenues for generating fate-committed Schwann cells using autologous cells that are effective in promoting functional recovery after trauma. 

SCLCs can be derived from adult non-neural tissue [4,8] by enhancing signaling pathways such as integrin and bFGF signaling [27]. However, such SCLCs suffer from phenotypic instability since the sequential activation of ErbB and Notch signaling pathways is required for the maturation of Schwann cells, and the stabilization of the Schwann cell phenotype is seldom addressed [11,12]. The direct addition of ErbB and Notch ligands to the medium is insufficient since the mechanical strain transmitted through the cell membrane also contributes to their activation. For this reason, contact with axons is the most efficient means to activate the signaling pathways that cement Schwann cell fate. In small animal models, rapid contact between transplanted SCLCs and axons is achieved due to small lesion size. In human patients with larger lesions, the regrowing axon fibers require time to reach the transplanted cells. This provides a higher chance for uncommitted SCLCs to spontaneously dedifferentiate into myofibroblasts. This not only reduces the number of functional Schwann cells to support therapeutic effects, but also causes fibrosis of the lesion site, further limiting rehabilitation.

In our pilot studies, rat DRG neurons were required to mediate the fate commitment of SCLCs obtained from hBMSC-derived neurospheres [11]. However, the risk of animal cell contamination of human cell products renders such protocols unacceptable for clinical use. In this study, we present a rapid single-step protocol to obtain highly pure cultures of hiSN from iPSCs to present the suitable signaling ligands to hBMSC-derived SCLCs for their commitment to Schwann cell fate. The purity and functional capacity of such Schwann cells was not different from those generated via co-culture with rat DRG neurons [12]. This was supported by immunocytochemical characterization with Schwann cell markers and demonstration of Schwann cell function by in vitro myelination (Figure 2). Results open avenues for a completely autologous route towards obtaining fate-committed Schwann cells. 

In sciatic nerve injury over a critical gap length, the inadequate recolonization of the lesion by supporting myelin-forming glial cells prevents spontaneous recovery [6,28]. Autograft and allograft, providing both physical guidance and Schwann cells to support regrowing axons, results in a high level of recovery. However, the clinical use of autografts is limited by donor site morbidity, and allografts are limited by availability and the need for immunosuppression to prevent host graft rejection. While immunosuppressants confer regenerative effect by limiting secondary injury caused by inflammatory response [29], they cause numerous side effects such as muscle weakness [30,31] and increased risk of opportunistic infections. Using Schwann cells derived from autologous non-neuronal cells combined with synthetic scaffolds to provide physical guidance for the regrowth of axons across the lesion [32] avoids these issues.

While endogenous Schwann cells can myelinate regrowing axons, their limited proliferative capacity and the time required for these cells to migrate into the scaffold results in insufficient recolonization of the gap within a defined time [33]. The lack of supply of neurotrophic factors and low degree of myelination by these endogenous Schwann cells results in stunted regeneration that is unable as support functional recovery [34,35], as observed in the Matrigel-only group, where only a thin bundle of regenerated axons was found in the conduit. The seeding of Schwann cells to the synthetic scaffold prior to transplantation not only guaranteed an adequate supply of Schwann cells, but also circumvented the need for cells to migrate into the scaffold. When such scaffolds were applied to rats with sciatic nerve transection, regeneration at both the histological level and behavioral rehabilitation was comparable to autograft. The fasciculation of the regenerated sciatic nerve in rats that received hBMSC-dSC transplant in a manner similar to intact nerves further implied an advanced stage of regeneration. The advent of this easily accessible source of fate-committed and potentially autologous Schwann cells, combined with on-going efforts improve the physical guidance properties of the conduit [36,37], promises robust regeneration across nerve gaps.

The SFI assessment used in this study is regarded as the gold standard for evaluating the repair of the sciatic nerve [17]. We further adopted ink-free computerized footprint analysis in this study (i) to reduce the anxiety of the animals when putting dye onto their feet and (ii) to avoid the smearing of footprints [38]. The significant improvement in SFI after the transplantation of hBMSC-dSCs indicated the recovery of sciatic nerve function. This result is in line with larger diameter of the regenerating nerve, implying an increase in the number of fibers connecting the two ends. At 12 weeks post-injury, variation in the compact myelination of the regenerated nerves reduced propagation synchrony due to differences in conduction velocity, causing the broadening of CMAP and sub-optimal muscle activation. Nonetheless, this initial outcome highlights the prospect for hBMSC-dSCs to achieve the uniform myelination of the regenerating sciatic nerve if allowed a longer recovery time, resulting in the resynchronization of CMAP in the sciatic nerve and the restoration of motor function. 

A similar reparative power of hBMSC-dSCs on CNS injury was revealed in the spinal cord contusion model. The apparent lack of recovery in the Schwann-cell-only groups (both hSC and hBMSC-dSC) was likely the result of the formation of an axon-restrictive CS-rich glial scar by astrocytes due to both injury and contact with Schwann cells [24,26]. Upon removal of this restrictive boundary via treatment with ChABC, axons ensheathed or myelinated by the grafted Schwann cells could be observed. The short observation period (12 weeks) limited the degree of functional recovery (in terms of BBB and foot fault scores), consistent with the findings of previous works by our group and other groups [7,25,26]. Nonetheless, rats that received peri-lesional Schwann cell transplant and ChABC injection demonstrated improvements in recovery over those which received ChABC but without accompanying Schwann cell transplant. A longer observation time will reveal if further recovery could be achieved. 

## 5. Conclusions

Schwann cells derived from hBMSCs achieved commitment to the Schwann cell fate following co-culture with hiSNs. The cells were functional in guiding axonal regrowth and remyelinating axons both in a bridged sciatic nerve model and a contused spinal cord model of adult rats. Significantly improved histological and functional recoveries were observed in the groups transplanted with hBMSC-dSCs. This work therefore primes translational research towards use of hBMSC-dSCs as therapeutic agents in both PNS and CNS trauma.

## Data Availability

Datasets of the current study are available from the corresponding authors upon request.

## Supplementary Materials

The following supporting information can be downloaded at: https://www.mdpi.com/article/10.3390/cells12111479/s1, Table S1. Immunocytochemical characterization of hBMSCs; Figure S1. Characterization of hBMSCs and primary rat DRG neurons; Figure S2. Representative phase-contrast images showing the morphologies of hBMSCs and hiPSCs at different stages of differentiation; Figure S3. Characterization of hBMSC-derived neural progenitor cells in neurosphere-like floating clusters; Figure S4. Characterization of hiPSCs and derived neurons; Figure S5. Co-culture of purified rat DRG neurons (Neurofilament) with hBMSC-dSCs (MAB1281, human nucleus); Figure S6. Outlined fascicles from Figure 6D; Figure S7. CSPG distribution in spinal cords of operated rats and effect of ChABC activity in CS digestion; Figure S8. Images of separate channels in Figure 7.
