# Supplementary material for: IPSC-Derived Sensory Neurons Directing Fate Commitment of Human BMSC-Derived Schwann Cells: Applications in Traumatic Neural Injuries
Source: Cells. 2023 May 25;12(11):1479. doi: 10.3390/cells12111479 (PMC10253081; doi:10.3390/cells12111479)
Supplement: Supplementary file 1 [file cells-12-01479-s001.zip › cells-2380942-supplementary.pdf]

Legends for supplementary table and figures

Table S1. Immunocytochemical characterisation of hBMSCs. (n = 5).

	**Marker**	**% of hBMSCs positive for the indicated marker (mean ± SD)**
Mesenchymal stem cell markers (n = 5)	CD73	98.98 ± 1.44
	CD90	98.35 ± 1.65
	CD105	95.08 ± 5.83
	STRO-1	97.38 ± 2.63
Neural stem cell marker (n = 5)	Nestin	58.16 ± 3.90
Hematopoietic cell marker (n = 5)	CD45	0.00 ± 0.00

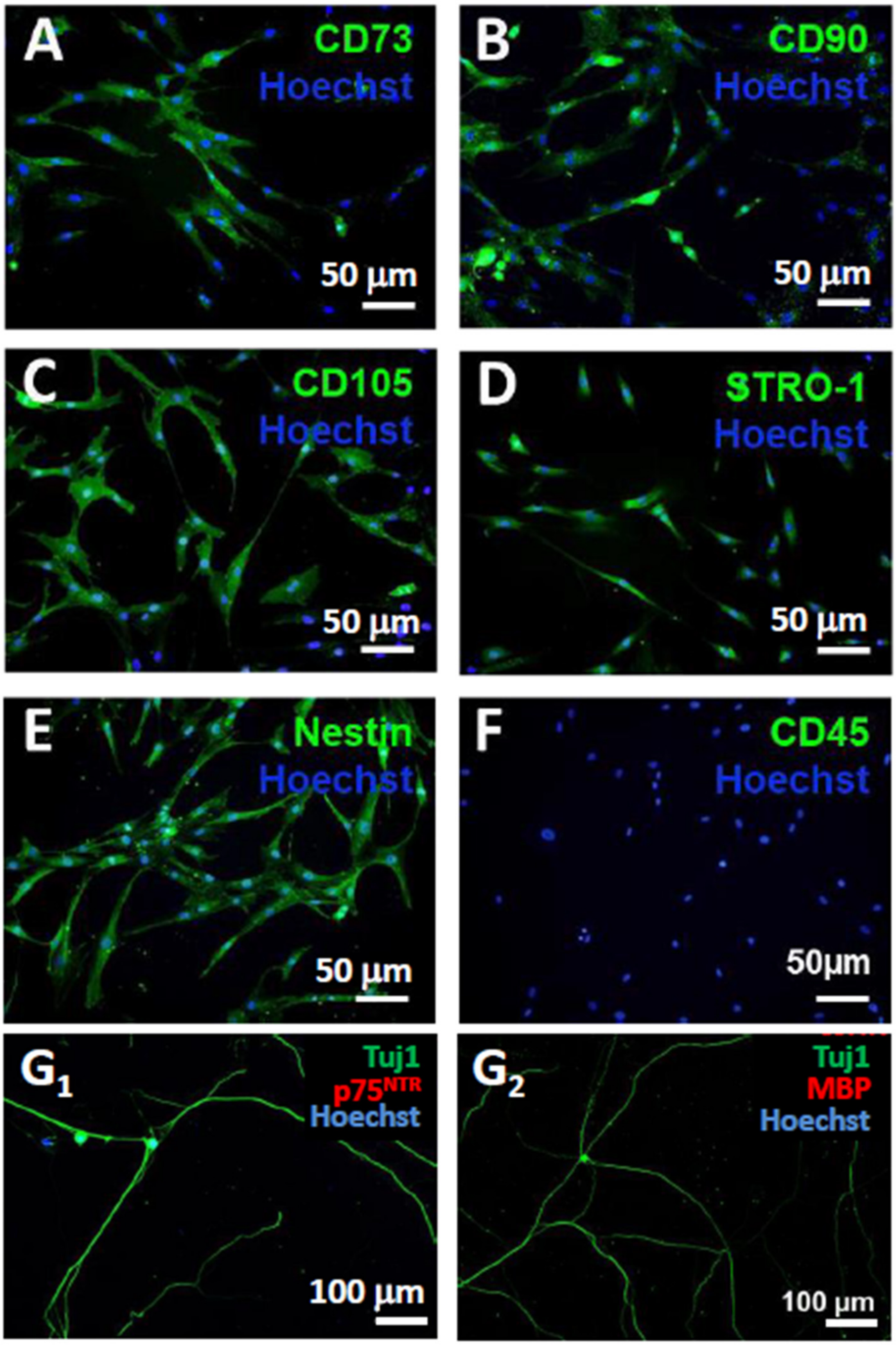

Figure S1. Characterisation of hBMSCs and primary rat DRG neurons. hBMSCs in passage 3 showed high percentage of positivity for mesenchymal stem cell markers (CD73 (**A**), CD90 (**B**), CD105 (**C**), STRO-1 (**D**)) and neural stem cell marker (Nestin (**E**)). Null staining of the haematopoietic cell marker CD45 (**F**) further demonstrated the purity of the culture. Rat DRG neuron culture cultures was free from contamination with host Schwann cells, indicated by p75NTR marker (**G1**) and no myelin segments left in the culture before seeding hBMSC-dSCs (**G2**). Scale bars: 50 µm (**A-F**), 100 µm (**G1, G2**).

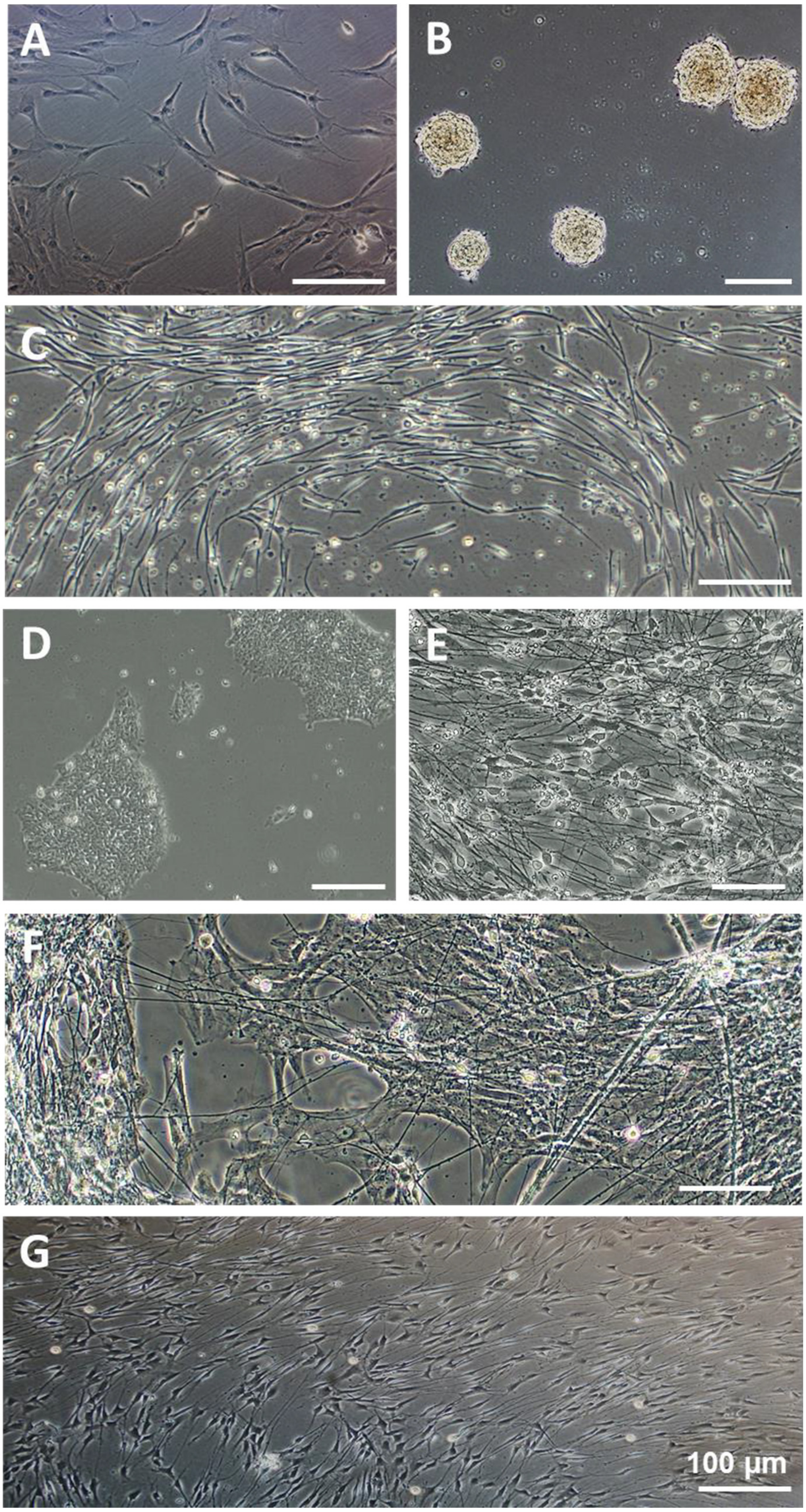

Figure S2. Representative phase-contrast images showing the morphologies of hBMSCs and hiPSCs at different stages of differentiation. Phase-contrast photomicrographs captured at different stages of differentiation of hBMSCs (hBMSCs at passage 3 (**A**), neurosphere-like cell clusters (**B**), spindle-like SCLCs (**C**)) and hiPSCs (hiPSC colonies (**D**), mature hiSN with multiple processes from the cell body (**E**)) show stage-specific, characteristic cell morphology. hBMSC-dSCLCs were co-cultured with hiSNs (**F**). Following removal of hiSNs by passaging, morphologically stable, lineage-committed SCLCs were obtained (**G**). Scale bars: 100 µm.

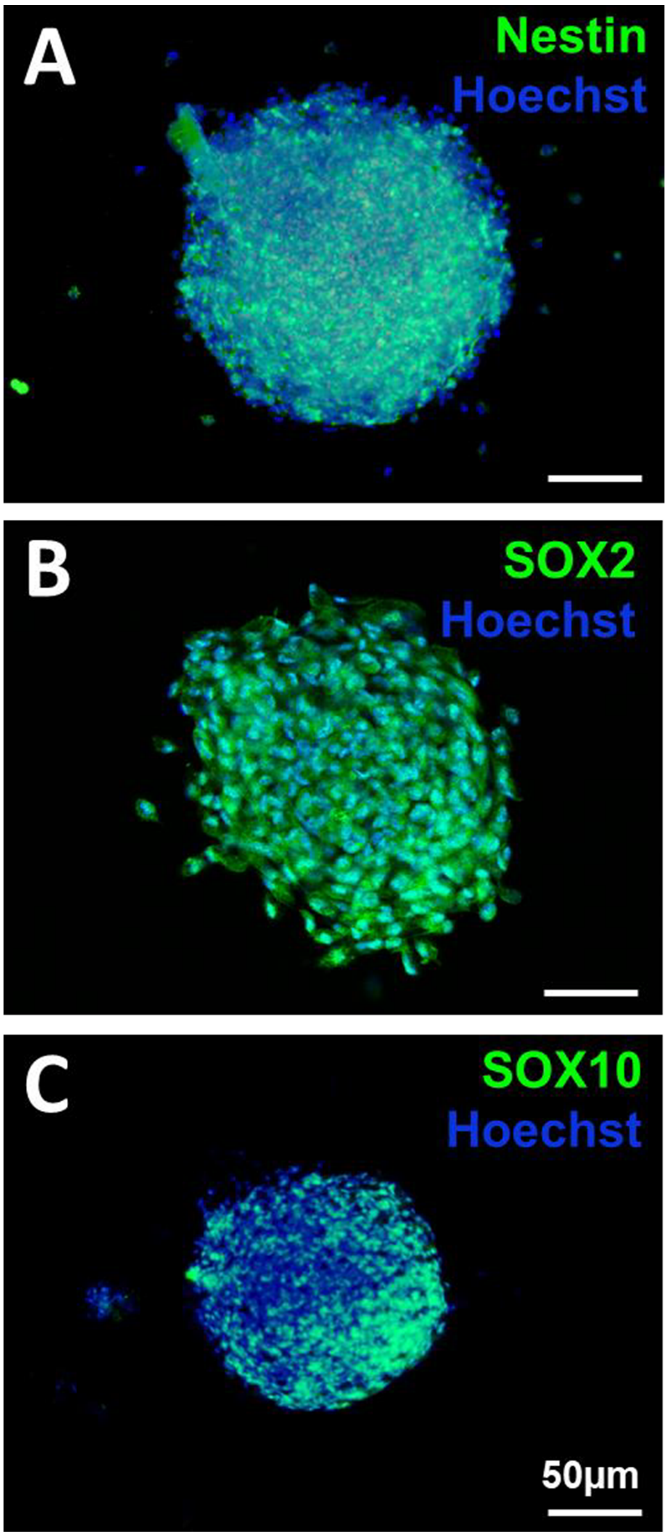

Figure S3. Characterisation of hBMSC-derived neural progenitor cells in neurosphere-like floating clusters. Neurosphere-like cell clusters were immunostained for neural stem cell markers (Nestin (**A**), SOX2 (**B**) and SOX10 (**C**)) to confirm the neural lineage potency of the cells. Scale bars: 50 µm.

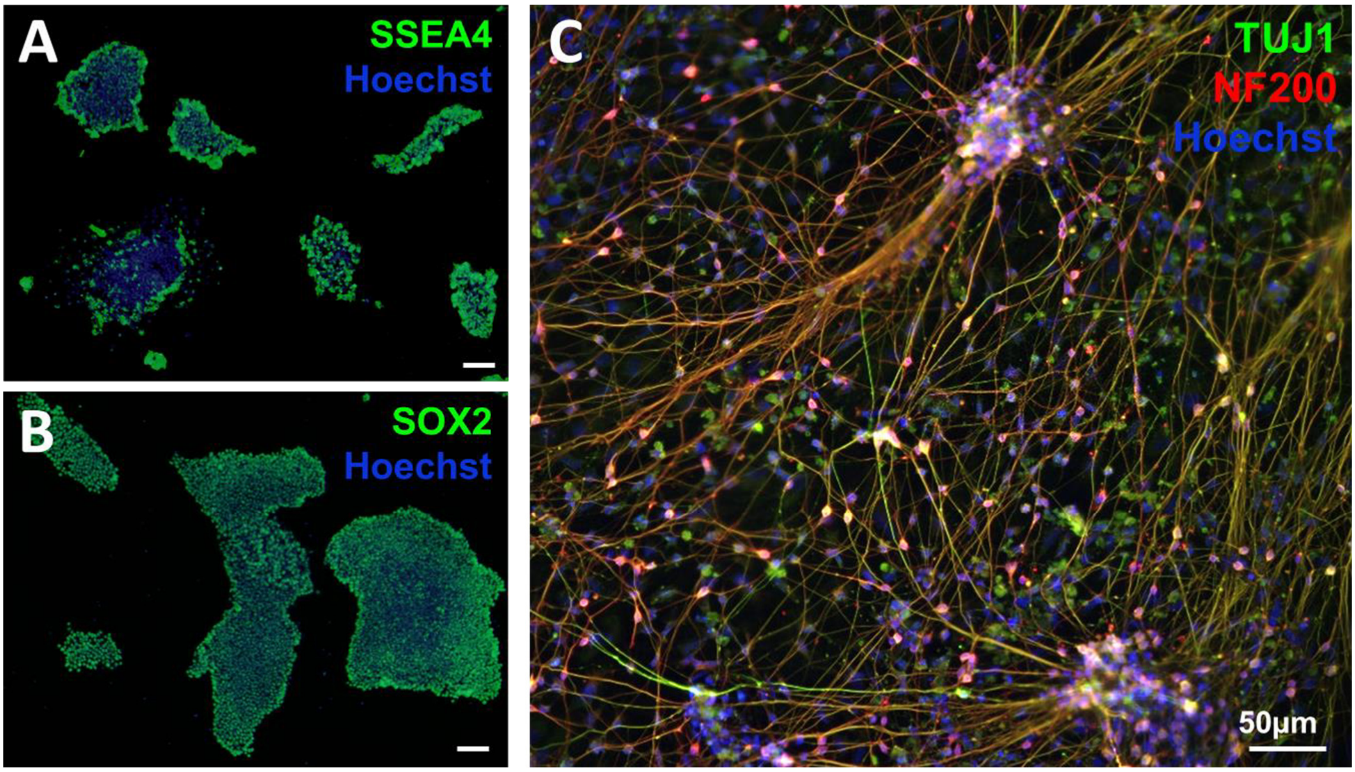

Figure S4. Characterization of hiPSCs and derived neurons. hiPSCs colonies were immunopositive for embryonic stem cell markers SSEA4 (**A**) and SOX2 (**B**). After the one-step induction, the cells possessed multiple processes and immunopositive to neuron specific tubulin type III (Tuj1) and NF200 (**C**). n = 3. Scale bars: 50 μm.

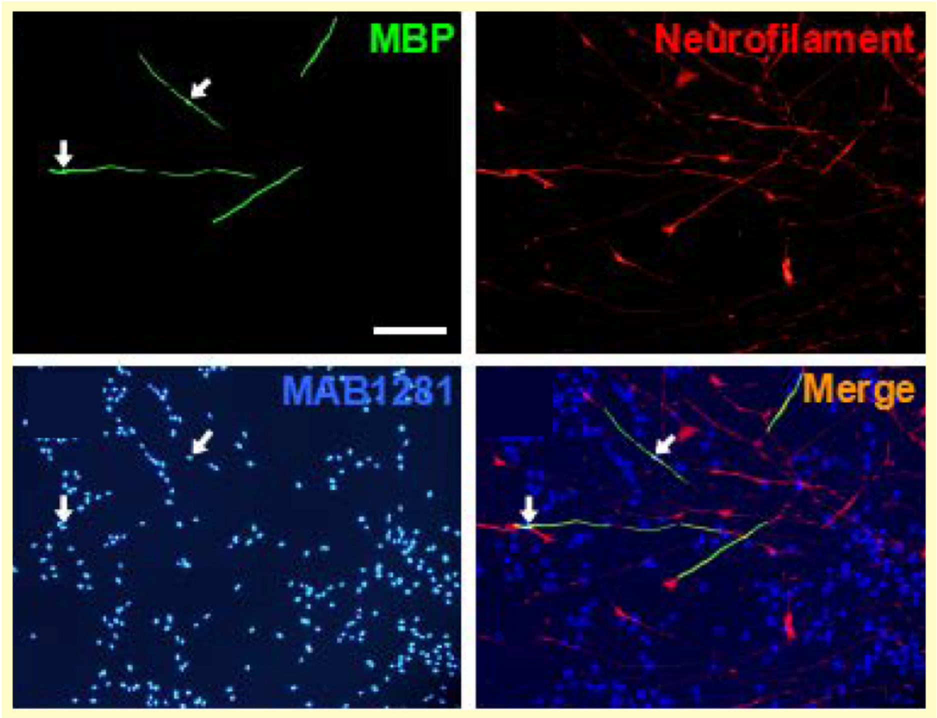

Figure S5. Co-culture of purified rat DRG neurons (Neurofilament) with hBMSC-dSCs (MAB1281, human nucleus). The hBMSC-dSCs myelinated the neurites resulting in MBP+ segments formed in the DRG network (White arrows: myelin segments). Scale bar: 100 μm.

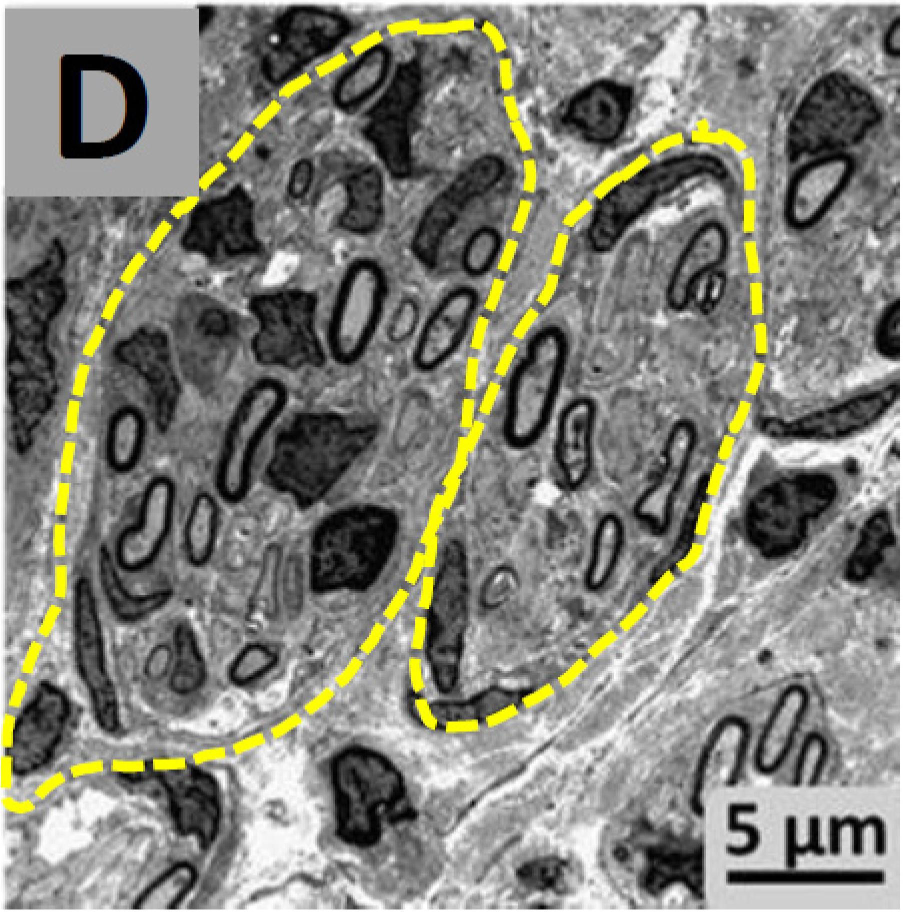

Figure S6. Outlined fascicles from Figure. 6 D1. Two fascicles are outlined with dotted yellow lines as an example to illustrate the calculation of axon cross sectional area. After manually identifying fascicle boundaries, the enclosed area was calculated automatically with ImageJ (NIH, USA).

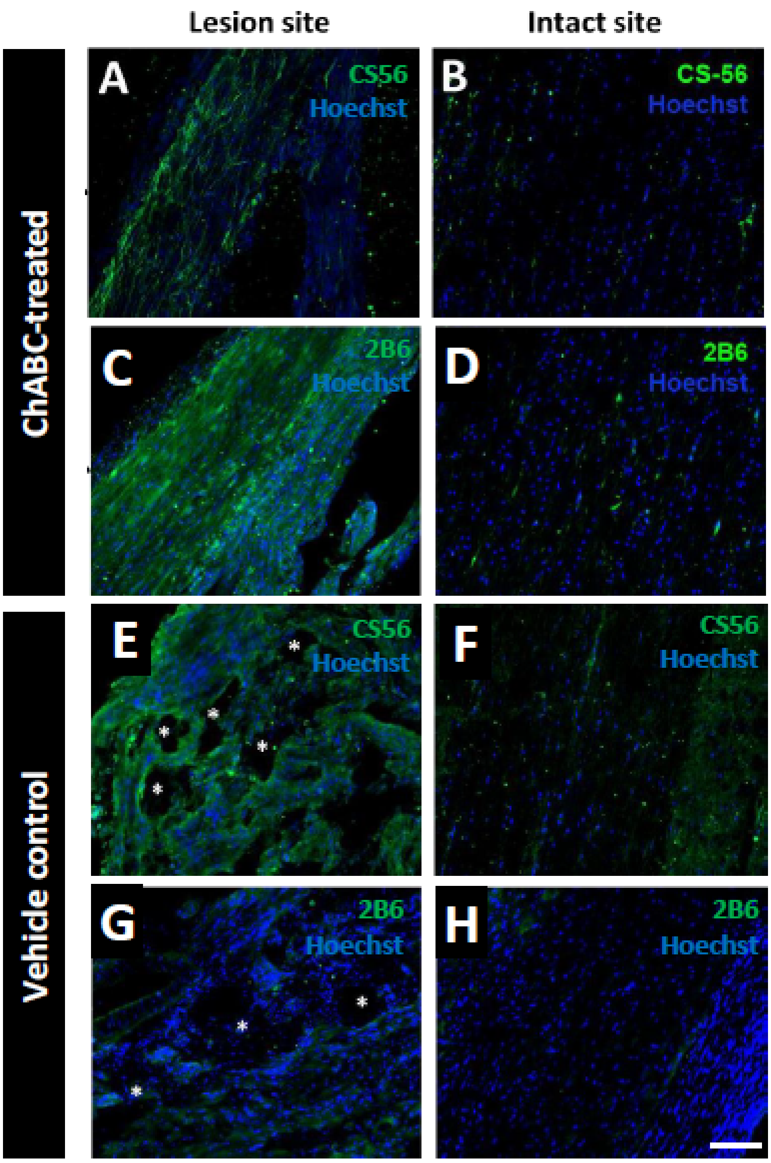

Figure S7. CSPG distribution in spinal cords of operated rats and effect of ChABC in CS digestion. CSPGs were indicated by CS-56 immunopositivity (**A,B,E,F**). The stub-epitopes exposed after digestion of CS by ChABC treatment were 2B6-positive. (**C,D,G,H**). *cystic. Scale bars: 100 µm, n=4.

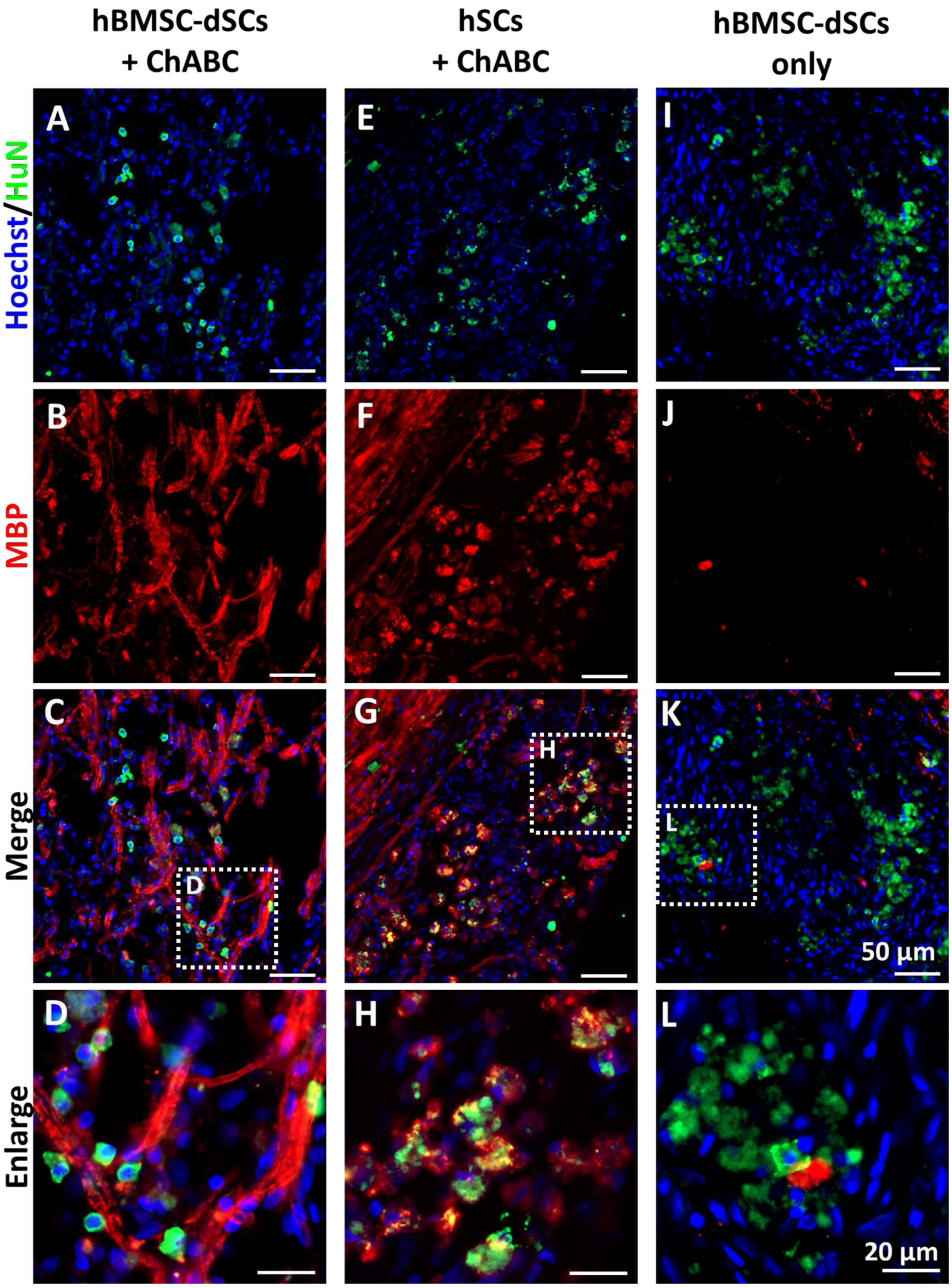

Figure S8. Images of separate channels in Figure 7. Scale bars for A–C, E–G & I–K: 50 μm; D, H,L: 20 μm.
